# Relationship between tinnitus and olfactory dysfunction: audiovisual, olfactory, and medical examinations

**DOI:** 10.3389/fpubh.2023.1124404

**Published:** 2023-04-20

**Authors:** Naomi Katayama, Tadao Yoshida, Tsutomu Nakashima, Yasuki Ito, Masaaki Teranishi, Takeshi Iwase, Saiko Sugiura, Kensuke Goto, Yasue Uchida, Yosuke Taki, Takafumi Nakada, Ai Tada, Hirokazu Suzuki, Yuta Nakano, Mariko Shimono, Naoki Saji, Anna Kogure, Emiko Shimizu, Michihiko Sone, Nobuyuki Hamajima

**Affiliations:** ^1^Department of Food Science, Nagoya Women's University, Nagoya, Japan; ^2^Department of Otorhinolaryngology, Nagoya University Graduate School of Medicine, Nagoya, Japan; ^3^Department of Rehabilitation, Ichinomiya Medical Treatment & Habilitation Center, Ichinomiya, Japan; ^4^Department of Otorhinolaryngology, Center for Sensory Organ, National Center for Geriatrics and Gerontology, Obu, Japan; ^5^Nagoya University, Nagoya, Japan; ^6^Department of Ophthalmology, Nagoya University Graduate School of Medicine, Nagoya, Japan; ^7^Department of Ophthalmology, Fujita Health University School of Medicine, Toyoake, Japan; ^8^Department of Otorhinolaryngology, National Hospital Organization Nagoya Medical Center, Nagoya, Japan; ^9^Department of Ophthalmology, Akita University Graduate School of Medicine, Akita, Japan; ^10^Toyota Josui Mental Clinic, Toyota, Japan; ^11^Department of Otolaryngology, Aichi Medical University, Nagakute, Japan; ^12^Department of Otorhinolaryngology, Nishichita General Hospital, Tokai, Japan; ^13^Center for Comprehensive Care and Research on Memory Disorders, National Center for Geriatrics and Gerontology, Obu, Japan; ^14^Department of Rehabilitation, National Center for Geriatrics and Gerontology, Obu, Japan; ^15^Department of Rehabilitation, Tokyo Medical and Dental University Hospital, Tokyo, Japan; ^16^Kishokai Medical Corporation, Nagoya, Japan

**Keywords:** health checkup, sensory dysfunctions, olfactory test, dietary habits, smoking, alcohol

## Abstract

**Introduction:**

Sensory dysfunctions and cognitive impairments are related to each other. Although a relationship between tinnitus and subjective olfactory dysfunction has been reported, there have been no reports investigating the relationship between tinnitus and olfactory test results.

**Methods:**

To investigate the relationship between tinnitus and olfactory test results, we conducted sensory tests, including hearing and visual examinations. The subjects included 510 community-dwelling individuals (295 women and 215 men) who attended a health checkup in Yakumo, Japan. The age of the subjects ranged from 40 to 91 years (mean ± standard deviation, 63.8 ± 9.9 years). The participants completed a self-reported questionnaire on subjective tinnitus, olfactory function, and hearing function, as well as their lifestyle. The health checkup included smell, hearing, vision, and blood examinations.

**Results:**

After adjusting for age and sex, the presence of tinnitus was significantly associated with subjective olfactory dysfunction, poor olfactory test results, hearing deterioration, vertigo, and headache. Additionally, high serum calcium levels and a low albumin/globulin ratio were significantly associated with low physical activity and nutrition. Women scored higher than men in olfactory and hearing examinations, but there was no gender difference in vision examinations.

**Conclusion:**

Subjective smell dysfunction and poor smell test results were significantly associated with tinnitus complaints. Hearing and vision were associated even after adjusting for age and sex. These findings suggest that evaluating the mutual relationships among sensory organs is important when evaluating the influence of sensory dysfunctions on cognitive function.

## Introduction

Many reports have described age-related sensory dysfunctions regarding vision, hearing, smell, and taste. However, there have been fewer studies investigating the relationships among these senses (1, 2). Oleszkiewicz et al. (3) reported alterations in gustatory sensitivity and taste preferences in individuals with blindness or deafness.

Not only a decrease but also abnormal sensations in smell and taste function are significant complaints in some patients after COVID-19 infection. Parosmia changes the usual perception of odors, such as when the smell of something familiar is distorted or when something that usually smells pleasant starts smelling foul. Phantosmia refers to an olfactory hallucination that is not caused by an actual odor. Parosmia and phantosmia can sometimes be more troublesome than hyposmia or anosmia. Tinnitus, on the other hand, is characterized by hearing sounds that are not present in the external environment. The relationship between phantosmia and smell dysfunction is similar to that between tinnitus and auditory dysfunction. Qualitative smell/taste disorders (such as phantosmia, parosmia, phantogeusia, and parageusia) have not yet been fully characterized, whereas quantitative disturbances (i.e., reduction/loss of smell/taste) have been widely investigated (4).

The coexistence of olfactory dysfunction and tinnitus with or without hearing loss after having COVID-19 has been reported (5–8). Some individuals did not complain of olfactory dysfunction but had abnormal results on smell tests after having a COVID-19 infection (9). Some individuals complained of the symptoms for over 1 year after having a COVID-19 infection (10, 11). The long-term prognosis is a matter that needs to be further examined in the future (12).

Park et al. (13) recently investigated the relationship between olfactory dysfunction and tinnitus in the general population, particularly in middle-aged and older adults. This study was based on a self-reported questionnaire completed by 25,534 people. Both olfactory function and tinnitus are associated with the limbic system, but few studies have clinically investigated the relationship. Moreover, there have been no reports comparing olfactory test results with tinnitus. In the present study, we attempted to investigate whether or not a relationship exists between olfactory test results and tinnitus, including hearing and vision tests.

## Methods

### Self-report questionnaire

We sent a detailed questionnaire, including questions on health problems and lifestyles, to the participants before the health examination. To the question “Can you smell?”, the participants chose one among the following four answers: (1) “sense smell well,” (2) “sometimes hard to smell,” (3) “slightly,” and (4) “not at all.” To the question “Do you have tinnitus?” the participants chose one among the three answers: (1) “no,” (2) “sometimes,” and (3) “always.” The difficulty of conversation includes two questions: “Can you hear one-to-one conversations without a hearing aid?” and “Can you hear conversations between four or five people without a hearing aid?” The participants selected one among four answers: (1) “completely,” (2) “mostly,” (3) “not much,” and (4) “almost nothing.” The participants also selected one among three answers, (1) “no,” (2) “sometimes,” and (3) “always,” to the question “Do you have vertigo?” We also asked them about the presence or absence of chronic headaches.

Dietary and lifestyle habits were investigated by the method described previously (14). Briefly, we investigated the frequency of 28 kinds of foods and drinks. (1) potatoes; (2) pumpkins; (3) carrots; (4) broccoli; (5) green leafy vegetables (green and yellow vegetables group A); (6) other green and yellow vegetables, including green peppers and snap beans (green and yellow vegetable group B); (7) cabbages; (8) radishes; (9) light-colored vegetables; (10) mushrooms; (11) seaweeds; (12) oranges and grapefruits; (13) other fruits, including strawberries, kiwis, apples, and watermelons; (14) tofus; (15) eggs; (16) chicken; (17) beef and pork; (18) hams, sausages, and bacons; (19) fish; (20) squid, shrimp, crabs, and octopuses; (21) shellfish; (22) deep-fried foods; (23) stir-fried foods; (24) miso soup; (25) milk; (26) yogurt; (27) green tea; and (28) coffee. In addition, we asked questions concerning sports and exercise per week as follows: rare, 1–2 h, 3–4 h, and 5 h or more. The answer “rare” was interpreted as having no exercise habits, while the other options were considered to have exercise habits (14).

We asked the participants about the amount of alcohol consumed with six kinds of beverages: beer, whiskey, wine, Japanese rice wine (Japanese sake), shochu (a traditional Japanese distilled spirit), and chuhai (a cocktail of shochu with fruit or soda). The amount of alcohol (g) per week was obtained from the table as follows: 20 g in 500 ml beer, 9.6 g in 30 ml whiskey single, 19.2g in 30 ml whiskey double, 11.5 g in a 120-ml glass of wine, 21.6 g in 180 ml (1 Gou) of Japanese sake, 36 g (180 ml) in a glass of shochu, and 14 g in 350 ml chuhai.^1^

The present status and history of smoking were enquired, including for heat-not-burn tobacco and electronic cigarettes (with or without nicotine). Then, we calculated the Brinkman index by multiplying the number of cigarettes smoked per day with the number of years of smoking while also incorporating the participants' conversion to the consumption of heat-not-burn tobacco. To achieve this, we utilized the questionnaire described in the Yakumo study (14, 15).

### Examination

We measured body weight, body mass index (BMI), and body fat percentage during the health checkup. We also conducted a blood test to assess the number of red and white blood cells, platelets, hemoglobin, hematocrit, mean corpuscular hemoglobin concentration (MCHC), total protein, albumin/globulin (A/G) ratio, globulin, triglyceride, high-density lipoprotein cholesterol, low-density lipoprotein cholesterol, hemoglobin A1c, creatinine, blood urea nitrogen, uric acid, serum calcium, aspartate aminotransferase: AST (GOT), alanine aminotransferase: ALT (GPT), alkaline phosphatase: ALP, C-reactive protein: CRP, and γ-glutamyl transpeptidase (γ GTP).

### Hearing test

Of the 510 participants, 369 of them underwent the hearing test. An otolaryngologist examined the ears, nose, and throat before the hearing test. In a quiet room, we evaluated hearing levels using an audiometer (Model AA-79S; Rion, Tokyo, Japan). The noise level in the examination room was measured every hour using a sound level meter (Rion NL-20; Rion) to confirm the room's suitability for the hearing test. The equivalent continuous sound level ranged from 41.1 to 53.9 dB, with an average of 45.0 dB. We measured the hearing level bilaterally at 1 kHz and 4 kHz. When the participants could respond to 30 dB at 1 kHz and 4 kHz, we determined the hearing test to be acceptable, either unilateral or bilateral.

### Olfactory test

Of the 369 people who underwent the hearing test, 298 hoped for the olfactory test. The Odor Stick Identification Test for Japanese (OSIT-J, Daiichi Yakuhin Sangyo, Tokyo, Japan) was developed as a simplified olfactory function test (16), which consists of 12 odors that are likely to be recognizable to Japanese individuals: Indian ink, wood, perfume, menthol, Japanese orange, curry, household gas, rose, Japanese cypress, sweaty socks, condensed milk, and roasted garlic. The participants were asked to identify the odor by choosing one of the six answers (one “correct,” three “incorrect,” and the other two were “unknown” and “not detected”). In this study, participants that recognized correctly six or more kinds of odors among the 12 kinds of odors were added to the good smell test group.

### Vision test

The best-corrected visual acuity (BCVA) was measured using an automatic vision tester (Nidek, NV-350, Gamagori, Japan) after the measurement of refractive errors (TONOREF III, Gamagori, Japan). The present study used visual acuities of 1.0 (20/20) and 0.8 (20/25) on either side or both sides to classify good or bad visual acuity.

### Statistical analysis

The Chi-square tests, Mann–Whitney tests, and generalized linear model analyses were performed. A logistic regression model estimated the odds ratio (OR) and 95% confidence intervals (CIs). The calculations were conducted using Stata 15 and SPSS 26.0, and statistical significance was set at a *p*-value of < 0.05.

### Ethics and consent

The Ethics Committee of Nagoya University School of Medicine approved this study (Approval number 2014–0207). All the participants approved the publication of anonymized data and provided informed consent to the participation.

## Results

### Participants in the health checkups

The participants were volunteers who attended an annual health examination in Yakumo, Hokkaido, Japan. These examinations were supported by the local government (the Yakumo study). In addition, a cross-sectional study was performed on 510 people (215 men and 295 women, average age: 63.8 years, age range 40–91 years) who attended a health checkup in 2019.

Table 1 shows the age distribution, subjective olfaction, smell test result, tinnitus, subjective hearing, hearing test result, and vision test result for women and men. In the age group of 80 or more people, two men were in their 90's. The age of men was significantly greater than that of women (Mann-Whitney test, *p* < 0.001). The questionnaire revealed that 25.4% and 8.8% of the participants reported experiencing tinnitus occasionally or constantly. The participants responded that subjective hearing in one-to-one conversations was much better than in conversations between four or five people. Two women and seven men used a hearing aid, and the answers were under status without a hearing aid.

**Table 1:** Results of questionnaire and tests associated with olfaction, tinnitus, hearing and vision.

		**Female**	**Male**	**Total**
Persons in each age group	40's	34 (12)	12 (6)	46 (9)
	50's	67 (23)	40 (19)	107 (21)
	60's	119 (40)	80 (37)	199 (39)
	70's	69 (23)	70 (33)	139 (27)
	80 or more	6 (2)	13 (6)	19 (4)
Age, median [25%tile, 75%tile]		65 [55, 69]	68 [60, 71]	65 [57, 71]
Can you smell?	Sense smell well	227 (77)	147 (68)	374 (73)
	Sometimes hard to smell	53 (18)	48 (22)	101 (20)
	Slightly	7 (2)	11 (5)	18 (4)
	Not at all	8 (3)	9 (4)	17 (3)
Smell test the number of correct answer to 12 kinds of odors	Six or more	151 (89)	86 (67)	237 (80)
	From three to five	16 (9)	31 (24)	47 (16)
	Two or less	2 (1)	12 (9)	14 (5)
Tinnitus	No	189 (64)	146 (68)	335 (66)
	Sometimes	86 (29)	44 (20)	130 (25)
	Always	20 (7)	25 (12)	45 (9)
^*^Can you hear one-to-one conversations?	Completely	206 (70)	131 (61)	337 (66)
	Mostly	81 (28)	76 (35)	157 (31)
	Not much	6 (2)	7 (3)	13 (3)
	Almost nothing	1 (0.3)	1 (0.5)	2 (0.4)
^*^Can you hear conversations between four or five people?	Completely	159 (54)	100 (47)	259 (51)
	Mostly	122 (42)	94 (44)	216 (43)
	Not much	11 (4)	18 (8)	29 (6)
	Almost nothing	1 (0.3)	2 (1)	3 (0.6)
Hearing test 1 kHz and 4 kHz 30 dB OK	Either side	205/230 (89)	85/166 (51)	290/396 (73)
	Bilateral	171/230 (74)	52/166 (31)	223/396 (56)
Vision test visual acuity 1.0 or more	Either side	188/292 (64)	131/211 (62)	319/503 (63)
	Bilateral	94/292 (32)	75/211 (36)	169/503 (34)
Vision test visual acuity 0.8 or more	Either side	241/292 (83)	164/211 (78)	405/503 (81)
	Bilateral	169/292 (58)	123/212 (58)	292/503 (58)

In the age group, there were two males in his 90's.

* Two women and seven men were using a hearing aid. The answers was under status without the hearing aid. Hearing test 30 dB was the smallest sound in the examination room. The values in parentheses are percentages.

### Influence of age and sex on smell, hearing, and vision

Table 2 shows the OR and 95% CI of age and sex when logistic regression analysis was performed. The explanatory variables were age and sex, and each objective variable is shown in Table 2. Both older age and male sex worsened the results in the smell and hearing tests. However, in the subjective evaluation of their olfactory and hearing functions, older age but not sex worsened the subjective evaluation. Men evaluated their smell and hearing functions as relatively good compared to women's evaluations.

**Table 2:** Influence of age and sex on subjective smell, smell test, subjective hearing, hearing test, visual acuity and tinnitus (logistic regression analysis).

**Objective variable**	**Explanatory variables**					
	**Age (years)**			**Sex (M** = **0, F** = **1)**		
	**Odds ratio**	***p***	**CI**	**Odds ratio**	***p***	**CI**
Subjective smell: sense smell well	0.954	< 0.0001	0.933–0.976	1.342	0.278	0.893–2.014
Smell test good (correct answer 6 or more)	0.928	< 0.0001	0.896–0.962	3.682	0.0001	1.969–6.920
One-to-one conversation subjective hearing complete	0.958	< 0.0001	0.939–0.978	1.315	0.1594	0.898–1.925
Between 4 or 5 persons subjective hearing complete	0.968	0.0006	0.950–0.986	1.219	0.2826	0.849–1.715
Hearing test 1 kHz and 4 kHz either side audible	0.901	< 0.0001	0.871–0.933	7.770	< 0.0001	4.481–13.475
Hearing test 1 kHz and 4 kHz both sides audible	0.907	< 0.0001	0.881–0.934	6.546	< 0.0001	4.034–10.620
Visual acuity 1.0 or more either side	0.934	< 0.0001	0.914–0.955	0.890	0.557	0.604–1.312
Visual acuity 1.0 or more both sides	0.940	< 0.0001	0.920–0.959	0.685	0.0614	0.460–1.018
Visual acuity 0.8 or more either side	0.925	< 0.0001	0.900–0.951	1.079	0.747	0.678–1.718
Visual acuity 0.8 or more both sides	0.949	< 0.0001	0.930–0.968	0.823	0.309	0.566–1.198
Tinnitus (+)	1.003	0.780	0.984–1.022	1.198	0.349	0.821–1.748

The visual acuity test result was worse in older age, but no significant sex difference was observed. In addition, the presence of tinnitus was not associated with age or sex.

### Factors associated with the smell test results

The influence of various factors on the smell test (correct answer: six or more) is shown in Table 3 before and after adjusting for age and sex. The subjective perception of good smell was significantly associated with good smell test scores. In addition, our analysis revealed a significant negative relationship between the presence of tinnitus and olfactory function. Specifically, participants with tinnitus had a significantly lower sense of smell, as evidenced by the results of the smell tests. However, vertigo, headache, subjective hearing, and hearing test results had no significant relationship with the smell test after we adjusted for age and sex. However, we found that the hearing test results for audibility at 4 kHz at 30 dB on either side were marginally close to reaching a significant level (*p* = 0.0664). Additionally, after adjusting for age and sex, the participants with a visual acuity of 0.8 or higher in both eyes had significantly higher scores on the smell test.

**Table 3:** Influence of various factors on smell test good.

**Factors**	**Unadjusted**			**Adjusted for age and sex**		
	**Odds ratio**	***p***	**95% CI**	**Odds ratio**	***p***	**95% CI**
Subjective smell: sense well	4.409	< 0.0001	2.425–8.016	3.588	0.0001	1.880–6.846
Tinnitus presence	0.521	0.0254	0.294–0.923	0.465	0.0168	0.248–0.871
Vertigo presence	1.329	0.4395	0.646–2.733	1.133	0.7559	0.515–2.497
Headache presence	1.880	0.3201	0.542–6.521	0.642	0.5376	0.157–2.630
One-to-one conversation subjective hearing complete	1.692	0.0746	0.949–3.017	1.312	0.3989	0.698–2.465
Between 4 or 5 people subjective hearing complete	1.441	0.2078	0.816-2.542	1.147	0.6635	0.619-2.126
Hearing test 1 kHz and 4 kHz 30 dB either side audible	3.865	< 0.0001	2.140–6.979	1.639	0.1571	0.827–3.251
Hearing test 1 kHz and 4 kHz 30 dB both sides audible	3.344	0.0001	1.852–6.038	1.353	0.3932	0.676–2.706
Hearing 4 kHz 30 dB either side audible	4.409	< 0.0001	2.425-8.016	1.909	0.0664	0.957-3.809
Hearing 4 kHz 30 dB both sides audible	3.351	0.0001	1.863–6.028	1.301	0.4615	0.646–2.623
Visual acuity 1.0 or more either side	2.248	0.0055	1.268–3.985	1.604	0.1520	0.840–3.064
Visual acuity 1.0 or more both sides	2.012	0.0288	1.075–3.765	1.610	0.1793	0.803–3.226
Visual acuity 0.8 or more either side	2.284	0.0153	1.172–4.452	1.347	0.4349	0.637–2.848
Visual acuity 0.8 or more both sides	2.898	0.0003	1.626–5.166	2.488	0.0060	1.299–4.766

### Factors associated with tinnitus

Table 4 shows the influence of various factors on the presence of tinnitus evaluated by logistic regression analysis before and after adjusting for age and sex. We observed a significant correlation between subjective good smell, performance on the smell test, and the absence of tinnitus complaints. Good subjective hearing and positive results of hearing tests were also associated with no tinnitus complaints. An elevated hearing threshold due to hearing loss at 1 kHz and 4 kHz was related to tinnitus. Visual acuity had no association with tinnitus, but vertigo and headache were significantly associated with tinnitus. Of the 43 people with chronic headaches, 22 reported having migraine-type headaches on one side, while 21 reported having non-migraine-type headaches. A logistic regression analysis revealed that migraine-type and non-migraine-type headaches were significantly associated with tinnitus. Table 4 depicts the blood examination results related to tinnitus. High serum calcium, globulin, and a low A/G ratio were associated with tinnitus.

**Table 4:** Influence of various factors on tinnitus presence.

**Factors**	**Unadjusted**			**Adjusted for age and sex**		
	**Odds ratio**	***p***	**95% CI**	**Odds ratio**	***p***	**95% CI**
Subjective smell: sense well	0.495	0.0006	0.331–0.741	0.474	0.0004	0.313–0.718
Smell test good	0.521	0.0254	0.294–0.923	0.461	0.0151	0.247–0.861
One–to–one conversation subjective hearing complete	0.469	0.0001	0.320–0.687	0.448	0.0001	0.303–0.664
Between 4 or 5 persons subjective hearing complete	0.362	< 0.0001	0.248–0.530	0.349	< 0.0001	0.237–0.514
Hearing level 1 kHz and 4 kHz 30dB either side audible	0.626	0.0438	0.396–0.987	0.504	0.0135	0.292–0.868
Hearing level 1 kHz and 4 kHz 30 dB both sides audible	0.704	0.0972	0.465–1.066	0.588	0.0362	0.357–0.966
Visual acuity 1.0 or more (both sides)	0.681	0.0603	0.455–1.017	0.676	0.0658	0.445–1.026
Visual acuity 1.0 or more (either side)	0.813	0.287	0.556–1.190	0.807	0.2916	0.543–1.202
Visual acuity 0.8 or more (both sides)	0.715	0.0780	0.493–1.038	0.711	0.0810	0.484–1.043
Visual acuity 0.8 or more (either side)	0.731	0.1780	0.464–1.153	0.715	0.1639	0.445–1.147
Vertigo presence	3.053	< 0.0001	1.992–4.680	3.045	< 0.0001	1.979–4.685
Headache presence	2.643	0.0026	1.405–4.975	2.717	0.0032	1.399–5.278
Current smoking	1.018	0.9444	0.608–1.705	1.090	0.754	0.637–1.863
Brinkman index	1.00016	0.4744	0.9997–1.0006	1.00037	0.1706	0.9998–1.0009
Alcohol intake (gram per week)	1.0007	0.238	0.9995–1.002	1.0008	0.234	0.9995–1.002
Having sports or exercise habits	0.711	0.078	0.487–1.039	0.703	0.0768	0.475–1.039
Serum calcium	1.924	0.0194	1.111–3.330	1.886	0.0242	1.086–3.274
Total protein	1.776	0.0160	1.113–2.835	1.748	0.0199	1.092–2.797
Globulin	1.842	0.0149	1.127–3.013	1.812	0.0201	1.098–2.991
Albumin/globulin (A/G) ratio	0.434	0.0347	0.200–0.942	0.444	0.0446	0.201– 0.981
Body mass index (BMI)	0.974	0.3147	0.925–1.025	0.977	0.388	0.928–1.030

Table 5 shows the relationship between tinnitus and exercise habits, including sports, in both women and men. Out of the 113 men who reported no exercise habits, 42 had tinnitus, while 27 out of 102 men who exercised regularly reported experiencing tinnitus. Although the proportion of tinnitus (+) was observed to be higher among those without exercise habits than in those with exercise habits, particularly in men, the difference was not statistically significant based on the chi-squared test (0.05 < *p* < 0.1). These findings are consistent with the trend observed in Table 4, which suggests that there is a potential relationship between having exercise habits and the absence of tinnitus (*p* = 0.0768).

**Table 5:** Relationship between tinnitus and exercise habits.

	**Exercise habits (–)**		**Exercise habits (**+**)**		
	**Female**	**Male**	**Female**	**Male**	**Total**
Tinnitus (−)	119	71	68	75	333
Tinnitus (+)	72	42	34	27	175
Total	191	113	102	102	508

### Hearing, visual, and olfactory dysfunctions: relationships, and the background

The relationship between the hearing test and the visual and olfactory tests is shown in Table 6. The good hearing result was associated with a good vision test after adjusting for age and sex, especially at 4 kHz rather than 1 kHz. Thus, impaired hearing and bad vision have some relationship with each other after adjusting for age and sex. In addition, we investigated factors associated with poor sensations. However, neither the hearing test result nor the vision test result had a significant relationship with BMI, hemoglobin A1c, triglycerides, cholesterol, the Brinkman index, the amount of alcohol intake, or the intake frequency of 28 kinds of food in this study.

**Table 6:** Relationship between hearing test and olfactory and vision tests (logistic regression analysis).

**Objective variables hearing**		**Explanatory variables**									
		**Factors**				**Age (years)**			**Sex (M** = **0, F** = **1)**		
		**Vision and smell**	**Odds ratio**	***p***	**CI**	**Odds ratio**	***p***	**CI**	**Odds ratio**	***p***	**CI**
Hearing 1 kHz and 4 kHz both sides audible	A	Visual acuity 1.0 or more both sides	1.760	0.0365	1.036–2.991	0.913	< 0.0001	0.886–0.940	7.024	< 0.0001	4.263–11.573
		Visual acuity 1.0 or more either side	1.816	0.0221	1.089–3.027	0.914	< 0.0001	0.887–0.941	6.795	< 0.0001	4.147–11.132
		Visual acuity 0.8 or more both sides	1.222	0.4299	0.742–2.014	0.909	< 0.0001	0.882–0.936	6.571	< 0.0001	4.033–10.707
		Visual acuity 0.8 or more either side	1.237	0.5015	0.665–2.302	0.908	< 0.0001	0.882–0.936	6.474	< 0.0001	3.985–10.519
		Smell test good	1.295	0.4682	0.644–2.602	0.908	< 0.0001	0.878–0.939	5.903	< 0.0001	3.331–10.464
Hearing 1 kHz and 4 kHz either side audible	B	Visual acuity 1.0 or more both sides	1.477	0.1992	0.814–2.679	0.906	< 0.0001	0.875–0.939	7.949	< 0.0001	4.555–13.874
		Visual acuity 1.0 or more either side	2.150	0.0074	1.227–3.767	0.912	< 0.0001	0.880–0.945	8.182	< 0.0001	4.653–14.387
		Visual acuity 0.8 or more both sides	1.714	0.0556	0.987–2.974	0.908	< 0.0001	0.876–0.940	8.078	< 0.0001	4.613–14.144
		Visual acuity 0.8 or more either side	1.823	0.0703	0.951–3.492	0.908	< 0.0001	0.877–0.940	7.772	< 0.0001	4.457–13.551
		Smell test good	1.643	0.1554	0.828–3.259	0.910	< 0.0001	0.876–0.945	5.891	< 0.0001	3.127–11.097
Hearing 1 kHz both sides audible	C	Visual acuity 1.0 or more both sides	1.499	0.2263	0.778–2.887	0.924	< 0.0001	0.892–0.958	1.762	0.0469	1.008–3.079
		Visual acuity 1.0 or more either side	0.994	0.9840	0.557–1.773	0.919	< 0.0001	0.887–0.953	1.708	0.0586	0.981–2.973
		Visual acuity 0.8 or more both sides	1.125	0.6844	0.638–1.984	0.921	< 0.0001	0.889–0.954	1.718	0.0562	0.986–2.995
		Visual acuity 0.8 or more either side	0.899	0.7578	0.456–1.772	0.918	< 0.0001	0.886–0.951	1.712	0.0575	0.983–2.981
		Smell test good	0.966	0.9303	0.446–2.095	0.912	< 0.0001	0.874–0.950	2.086	0.0360	1.049–4.149
Hearing 1 kHz either side audible	D	Visual acuity 1.0 or more both sides	2.500	0.2439	0.535–11.672	0.870	0.0002	0.809–0.937	2.818	0.0647	0.939–8.456
		Visual acuity1.0 or more either side	3.910	0.0241	1.195–12.787	0.878	0.006	0.815–0.945	2.836	0.0650	0.937–8.583
		Visual acuity 0.8 or more both sides	2.549	0.0989	0.839–7.743	0.872	0.0003	0.810–0.940	2.816	0.0656	0.936–8.474
		Visual acuity 0.8 or more either side	2.007	0.1906	0.707–5.700	0.869	0.0002	0.807–0.935	2.667	0.0800	0.889–8.001
		Smell test good	1.248	0.708	0.391–3.985	0.873	0.0003	0.811–0.940	2.867	0.0932	0.838–9.801
Hearing 4 kHz both sides audible	E	Visual acuity 1.0 or more both sides	1.749	0.0434	1.017–3.007	0.912	< 0.0001	0.885–0.940	8.221	< 0.0001	4.941–13.677
		Visual acuity 1.0 or more either side	2.325	0.0017	1.372–3.940	0.916	< 0.0001	0.888–0.944	8.329	< 0.0001	4.990–13.902
		Visual acuity 0.8 or more both sides	1.363	0.2333	0.819–2.270	0.909	< 0.0001	0.882–0.937	7.8011	< 0.0001	4.733–12.858
		Visual acuity 0.8 or more either side	1.465	0.2355	0.780–2.752	0.909	< 0.0001	0.882–0.937	7.635	< 0.0001	4.648–12.542
		Smell test good	1.252	0.5296	0.620–2.532	0.909	< 0.0001	0.878–0.940	7.176	< 0.0001	3.997–12.883
Hearing 4 kHz either side audible	F	Visual acuity 1.0 or more both sides	1.262	0.4484	0.692–2.302	0.906	< 0.0001	0.874–0.939	8.302	< 0.0001	4.684–14.714
		Visual acuity 1.0 or more either side	1.796	0.0432	1.018–3.167	0.911	< 0.0001	0.879–0.944	8.477	< 0.0001	4.763–15.088
		Visual acuity 0.8 or more both sides	1.569	0.1147	0.896–2.747	0.908	< 0.0001	0.876–0.941	8.483	< 0.0001	4.771–15.083
		Visual acuity 0.8 or more either side	1.931	0.0501	1.000–3.731	0.91	< 0.0001	0.878–0.943	8.312	< 0.0001	4.678–14.770
		Smell test good	1.922	0.0639	0.963–3.835	0.911	< 0.0001	0.876–0.947	6.502	< 0.0001	3.359–12.585

Objective variable A; hearing 1&4 kHz both sides OK, B; hearing 1&4 kHz either side OK, C; hearing 1 kHz both sides OK, D; hearing 1 kHz either side OK, E; hearing 4 kHz both sides OK, F; hearing 4 kHz either side OK.

We investigated the influence of diabetes mellitus (DM), hypertension, and dyslipidemia on visual, olfactory, and auditory dysfunctions from the medical history, the drug intake, and the blood examination results, including hemoglobin A1c, triglyceride, total cholesterol, HDL, and LDL cholesterol. The logistic regression analysis revealed that medical history of DM (OR = 1.97, 95% CI, 1.05–3.68) and anti-DM drug intake (OR = 2.08, 95% CI, 1.036–4.170) but not hemoglobin A1c had a significant relationship with visual acuity <1.0 on both sides after adjusting for age and sex. However, we could not find the influence of DM on olfactory and hearing test results. We could not find the influence of hypertension and dyslipidemia on the results of the visual, olfactory, and hearing tests. Table 6 shows significant associations between hearing and visual acuity test results, even after controlling for other explanatory variables such as medical history and drug intake for DM, hypertension, and dyslipidemia.

We investigated the effects of smoking on olfactory, hearing, and vision tests. Table 7 reveals the smoking situation. Of the current smokers, 13 consumed heat-not-burn tobacco, and three consumed electronic cigarettes with nicotine. After adjusting for age and sex, a logistic regression analysis was conducted to evaluate the association between smoking status (never, quit, and current smoker) or the Brinkman index and test results. The results showed that only the vision test was significantly associated with a Brinkman index of 100 or higher, with a visual acuity <1.0 on both sides (*p* = 0.0469) and of <0.8 on both sides (*p* = 0.0230). However, we found no significant relationship between alcohol consumption and test results for visual, olfactory, and auditory perception after adjusting for age and sex.

**Table 7:** Smoking situation in participants.

	**Female (*****n*** = **295)**			**Male (*****n*** = **213)**		
	**Never**	**Quit**	**Current smoker**	**Never**	**Quit**	**Current smoker**
Number of persons	203 (68.8%)	66 (22.4%)	26 (8.8%)	48 (22.5%)	116 (54.5%)	49 (23.0%)
Age	65 [58, 71]	61.5 [47, 67]	58 [52, 67]	70 [60.5, 76.5]	69 [62, 71]	62 [55, 68]
Brinkman Index	0	153 [60, 395]	512.5 [280, 760]	0	540 [300, 800]	760 [560, 1000]
Age at smoking initiation		20 [20, 23]	20 [20, 25]		20 [20, 20]	20 [20, 20]
Age at quitting smoking		40 [30, 50]			45 [40, 56]	
Body Mass Index (BMI)	22.8 [20.6, 25.4]	22.8 [20.2, 26.5]	22.15 [20.9, 23.5]	24.4 [22.15, 26.9]	24.6 [22.3, 26.4]	24.3 [21.5, 25.7]

Figures are median [25th percentile, 75th percentile].

**Figure 1:**
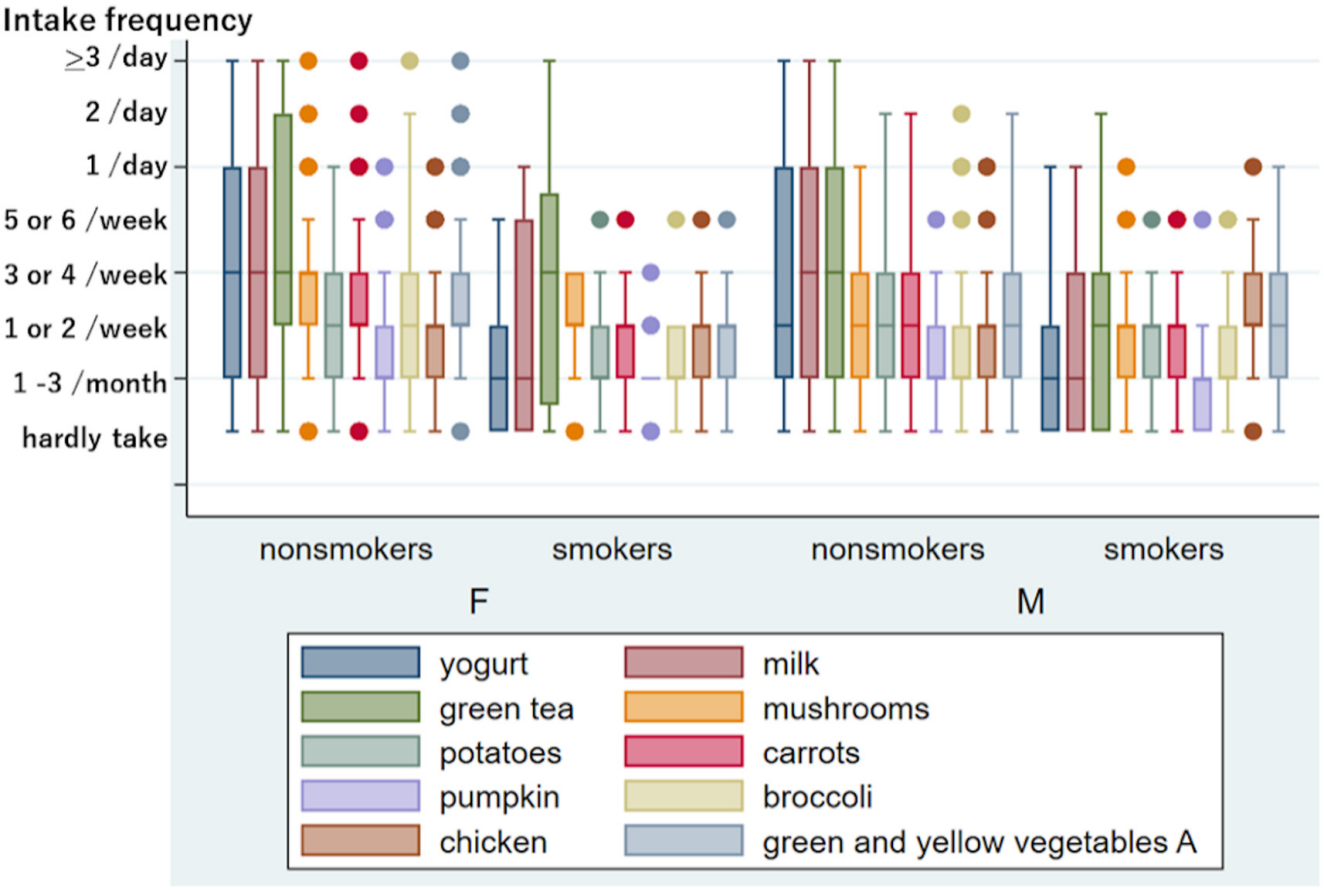
Frequency of eating and drinking in non-smokers and current smoker among women and men. Ten kinds of food and drink with a significant difference between non-smokers and current smokers after adjusting for age in women and/or men. The y-axis shows the intake frequency divided into 8. If the median intake frequency is within the lower quartile, the box's bottom line is thicker than its top line. Similarly, the box's top line is thicker than its bottom line if the median intake frequency is within the upper quartile. Outliers indicated by dots include multiple individuals. The intake frequency and the number of persons indicated by words are as follows: in 269 female nonsmokers, mushrooms “hardly take”: five, “1/day”: 26, “2/day”: three, “>3/day”: one; carrots “hardly take”: seven, “1/day”: 13, “2/day”: one, “>3/day”: one; pumpkin “5 or 6/week”: five, “1/day”: one; broccoli “>3/day”: one; chicken “5 or 6/week”: eight, “1/day”: two; green and yellow vegetables A “hardly take”: two, “1/day”: 19, “2/day” five, “>3/day”: one. In 26 female smokers, mushrooms “hardly take”: two; potatoes “5 or 6/week”: one; carrots “5 or 6/week”: one; pumpkin “hardly take”: six, “1 or 2/week”: four, “3 or 4/week”: one; broccoli “5 or 6/week”: one; chicken “5 or 6/week”: one; green and yellow vegetables A “5 or 6/week”: one. In 166 male nonsmokers, pumpkin “5 or 6/week”: one; broccoli “5 or 6/week”: two, “1/day”: three, “2/day”: one; chicken “5 or 6/week”: three, “1/day”: three. In 49 male smokers, mushrooms “5 or 6/week”: three, “1/day”: one; potatoes “5 or 6/week”: one; carrots “5 or 6/week”: four; pumpkin “5 or 6 week”: one; broccoli “5 or 6/week”: four; chicken “hardly take”: one, “1/day”: one.

**Figure 2:**
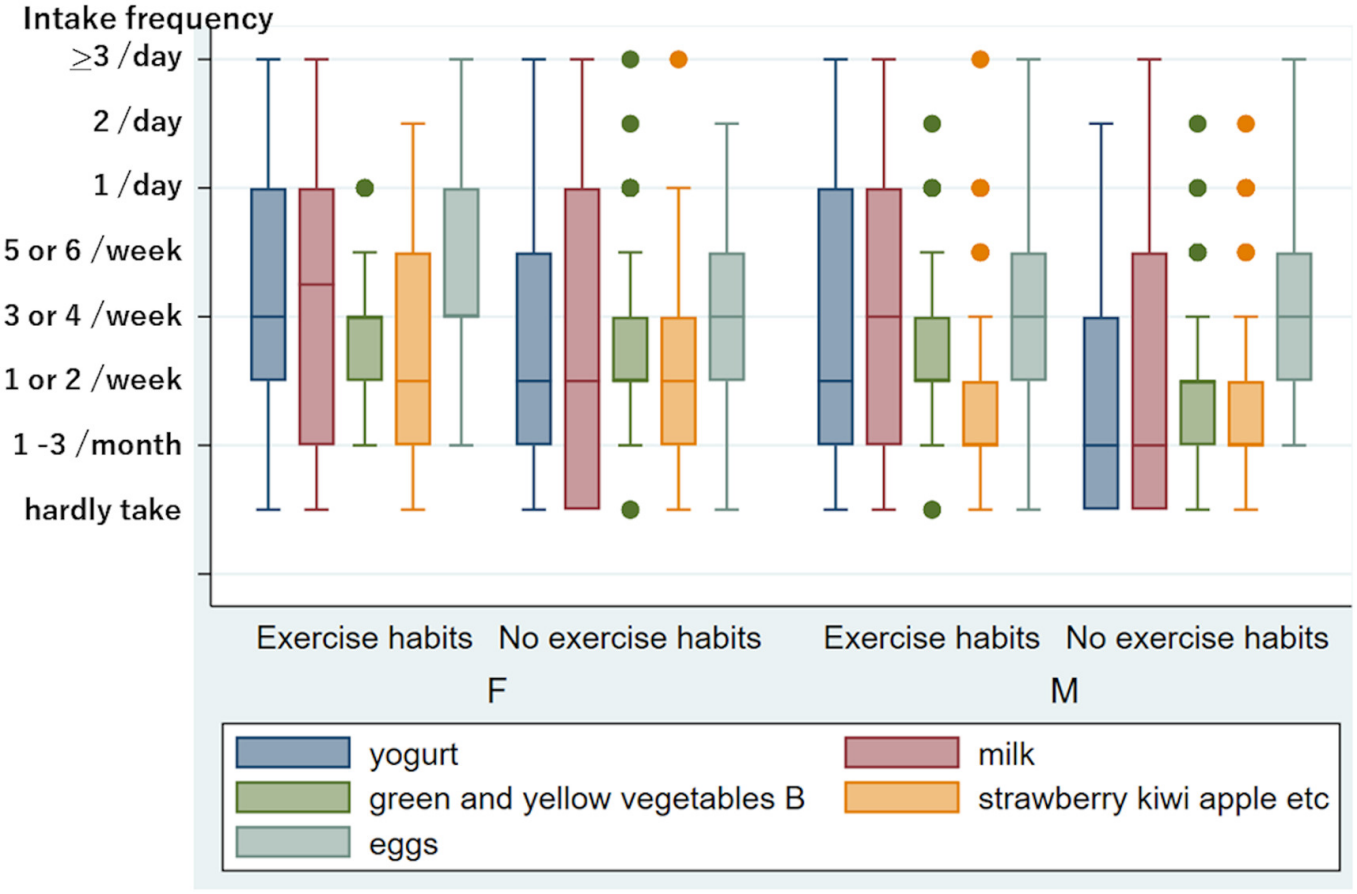
Frequency of eating and drinking in people with and without exercise habits in women and men. Five kinds of food and drink with a significant difference between persons with and without exercise habits after adjusting for age in women and/or men. The y-axis is the same as in Figure 1. The number of people belonging to outliers is as follows: In 102 women with exercise habits, green and yellow vegetables B “1/day”: five. In 192 women without exercise habits, green and yellow vegetables B “hardly take”: three, “1/day”: 13, “2/day”: one, “>3/day”: one; strawberry, kiwi, apple, etc. “>3/day”: one. In 102 males with exercise habits, green and yellow vegetables B “hardly take”: two, “1/day”: four, “2/day”: one; strawberries, kiwis, apples, etc. “5 or 6/week”: five, “1/day”: five, “>3/day”: one. In 113 men without exercise habits, green and yellow vegetables B “5 or 6/week”: six, “1/day”: two, “2/day”: one; strawberries, kiwis, apples, etc. “5 or 6/week”: two, “1/day”: six, “2/day”: one.

Figure 1 shows the difference in eating and drinking habits between non-smokers and current smokers. The Mann–Whitney test revealed that the current smoking status was significantly associated with a low intake frequency of vegetables (carrots, *p* < 0.0001, broccoli, *p* = 0.0011), dairy products (yogurt and milk, *p* < 0.0001), and green tea, *p* = 0.0002) in total. Thus, smoking habits reduced healthy eating and drinking patterns significantly. No exercise habits were also significantly associated with a low intake of dairy products (yogurt *p* < 0.0001, milk *p* = 0.0001), vegetables (green and yellow vegetable group B, *p* = 0.0051), and fruits (strawberries, kiwis, apples, etc., *p* = 0.0005) (Figure 2). However, we could not find a significant influence of food and drink intake frequency on tinnitus and sensory dysfunctions after adjusting for age and sex.

## Discussion

We found a relationship between tinnitus and poor olfactory test results after adjusting for age and sex. The findings are consistent with those of a previous report based on questionnaire responses (13). Because the subjective evaluation of sensory functions differed from the sensory test results (17–20), we compared the presence of tinnitus and olfactory test results. The relationship between tinnitus and the limbic system has been studied using neuroimaging techniques, including positron emission tomography (PET), functional MRI, and voxel-based morphometry (21–23). The olfactory function connects with the limbic system, including the hippocampus (24–26). Therefore, tinnitus and olfactory dysfunction may have a strong association with the limbic system and are also associated with depression (27, 28). Sound therapy for tinnitus may work by altering limbic and auditory networks (29). Olfactory training to restore the olfactory function was associated with increased gray matter volume of the hippocampus and the thalamus (30). Thus, rehabilitation aimed at improving both tinnitus and olfactory dysfunction can stimulate the senses.

Research has shown that individuals can experience auditory, vestibular, olfactory, and gustatory dysfunctions for an extended time following a COVID-19 infection (31). Additionally, numerous literature reviews have demonstrated that long-term COVID is frequently associated with tinnitus and olfactory and gustatory dysfunction (32). These findings are consistent with the results of our report.

**Figure 3:**
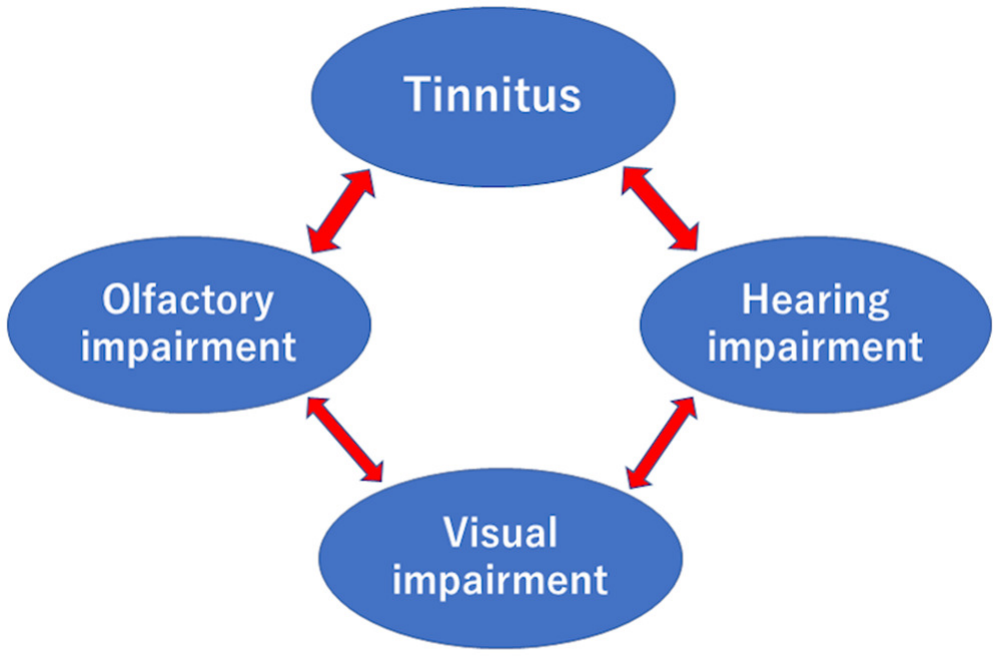
Relationships between tinnitus, olfactory, hearing, and visual impairments after adjusting for age and sex. Arrows were recognized in this study.

However, the present study did not reveal a relationship between hearing and olfaction after adjusting for age and sex. However, tinnitus and auditory dysfunction were significantly associated after adjusting for age and sex. The connection between hearing loss and tinnitus is widely known. For example, vertigo, hearing loss, and tinnitus are significant symptoms of Meniere's disease (33), and tinnitus accompanying idiopathic sudden sensorineural hearing loss is an important symptom for the treatment (34, 35). In addition, we recognized a relationship between visual acuity and hearing or smell after adjusting for age and sex. Figure 3 demonstrates these mutual relationships. The entorhinal cortex, the primary interface between the hippocampus and neocortex, may be related to the mutual relationships because it relates to audiovisual information processing and tinnitus (36).

High serum calcium and a low A/G ratio were identified as risks for tinnitus in this study. The risk may be related to physical activity and nutrition. We could not observe a statistically beneficial effect of exercise on tinnitus (0.05 < *p* < 0.1); however, several reports have revealed that physical activity significantly reduced the risk of tinnitus (37, 38). Because exercise can cause a decrease in serum calcium (39, 40), exercise habits that lower the serum calcium level may be better for reducing tinnitus. Dawes et al. (41) reported that higher calcium intake was associated with an increased risk of tinnitus. As smoking and exercise habits significantly influence dietary habits (42), as shown in Figures 1, 2, the influence of lifestyle habits should be investigated comprehensively. In addition, olfactory dysfunction may be related to food choices and eating enjoyment.

We did not observe a relationship between hearing loss and diabetes mellitus after adjusting for age and sex. Previous studies have reported a significant association between hearing loss and diabetes mellitus (43–46), but some did not (47, 48). Similarly, we did not observe a relationship between olfactory dysfunction and diabetes mellitus. Numerous studies have reported a relationship between olfactory dysfunction and diabetes mellitus; however, some of them did not find such a relationship (49). Naka et al. (50) found no significant difference in smell function between healthy subjects and patients with uncomplicated diabetes mellitus.

The history of hypertension was independently associated with a modestly higher risk of hearing loss (multivariable-adjusted relative risk, 1.04 [1.01–1.07]) (51). Higher systolic blood pressure was associated with the incidence of retinopathy (hazard ratio per 10 mmHg, 1.15 [1.07–1.20]) in multivariate analysis (52). Some signs of hypertensive retinopathy, specifically generalized and focal retinal arteriolar narrowing, may be subclinical indicators of hypertension and may be detected even before the clinical diagnosis (53). Considering the risk ratio and the relationship between hypertensive retinopathy and the clinical diagnosis of hypertension, at least the number of participants should have been greater to reveal a significant difference in the hearing and vision tests between participants with and without hypertension after adjusting for age and sex.

Many reports have described the influence of smoking on sensory functions, including olfactory (17), visual (54, 55), and hearing impairments (56, 57). While the present study found an association between smoking and visual impairments, no significant relationship was observed between smoking and olfactory or auditory dysfunctions. Although Nomura et al. (58) reported that a Brinkman index of 750 or higher was related to hearing loss in a Japanese operating company, there was no significant relationship between a Brinkman index of 750 or higher and hearing after adjusting for age and sex in the present study. Smoking has also been reported to be associated with tinnitus (59, 60). However, we could not find a relationship between tinnitus and current smoking or the Brinkman index. The negative results may depend on a characteristic of our study group, in which 36% of participants quit smoking. Typically, cigarette smokers have a lower BMI than non-smokers after controlling for age in men and women (61), but there was no significant difference in BMI observed between smokers and non-smokers in this study.

Hearing aids are helpful to compensate for hearing loss, but the percentage of hearing aid users (9/510) was low during this health checkup. The low percentage may be related to the fact that the nearest hearing aid store is ~100 km away. Many reports have described the effects of sensory dysfunctions on cognitive impairment or dementia. For example, the effects of olfactory, hearing, and visual impairment have been investigated (62). In addition, dual or multiple sensory impairments may synergistically influence cognitive impairment (63, 64). A few studies have reported a background of dual or multiple sensory impairments (65, 66). The present study revealed the results of the visual acuity test, but neither the olfactory nor the hearing tests were related to smoking habits after adjusting for age and sex. Further studies should investigate the mutual relationships among sensory dysfunctions.

The limitation of this study was the relatively small sample size, and not all participants underwent sensory testing, as their participation in the health checkup was voluntary. Additionally, the participants in this study may have had higher health consciousness compared to the general population of Japan, as evidenced by the high proportion of individuals who had quit smoking (more than 70%) (67).

## Conclusion

To date, the relationship between tinnitus and olfactory test results has not been investigated, but a significant association between tinnitus and subjective olfactory dysfunction has been reported. We found a significant relationship between tinnitus and olfactory test results after adjusting for age and sex. In the present study, hearing and olfactory impairments were associated with visual impairment after adjusting for age, sex, and other confounding factors. Various studies have reported the persistence of tinnitus and olfactory and gustatory dysfunctions in people with long-term COVID-19. Mutual relationships among sensory dysfunctions need to be studied in future studies.

## Data availability statement

The original contributions presented in the study are included in the article/Supplementary material, further inquiries can be directed to the corresponding author.

## Ethics statement

The studies involving human participants were reviewed and approved by the Ethics Committee of Nagoya University School of Medicine (approval number 2014–0207). The patients/participants provided their written informed consent to participate in this study.

## Author contributions

NK, TY, TsN, and MiS designed this study. NK, TY, and TaN performed olfactory tests and evaluations. TY, TaN, AK, and ES performed hearing tests and evaluations. YI, TI, KG, YT, AT, and YN performed vision tests and evaluations. TY, TsN, MT, SS, YU, HS, MaS, and NS evaluated multiple sensory dysfunctions and related factors. TY, TsN, and NH performed the statistical analysis. The manuscript was written by NK, TY, TsN, YI, MT, and NH. All authors contributed to the article and approved the submitted version.

## Footnotes

^1^ https://www.kirin.co.jp/csv/arp/proper/check_list.html
